# Pennsylvania State Veterinary Medical Association

**Published:** 1901-05

**Authors:** 


Secretary.

### PENNSYLVANIA STATE VETERINARY MEDICAL ASSOCIATION.

(Continued from page 271.)

Corresponding Secretary E. M. Ranck made a written report. Of special interest was the paper received from Vice-President-elect Theodore Roosevelt in reference to veterinarians of the army, which was received and filed.

*Reports of County Secretaries.*—Dr. George B. Johnson moved that the reports of County Secretaries be taken up alphabetically, which was adopted, and they were made as follows:

Adams Co., Dr. M. Moriarity reported hog-cholera and forage-poisoning.

Bucks Co., W. G. Benner reported several cases of hydrophobia in cattle, hog-cholera, and swine-plague.

Delaware Co., W. H. Mattson spoke of a few isolated cases of tetanus, forage-poisoning, and hydrophobia, and called attention to some faults in the Inter-State tests for tuberculosis.

Erie Co., J. B. Irons reported.

Franklin Co., J. Bradley made a verbal report that there were no contagious diseases of any importance.

Lancaster Co., B. F. Bachman had moved from Lancaster Co. to Allegheny, so was not familiar with the diseases in the former county. Dr. Joseph Johnson reported from this county a few cases of contagious ophthalmia and hog-cholera, but nothing serious.

Lebanon Co., M. J. Collins reported hog-cholera, contagious abortion in cows, many more cases of azoturia and tetanus than former years, parturient apoplexy, and he thinks tuberculosis among dairy cattle is increasing.

Luzerne Co., J. H. Timberman read an interesting report on the diseases in this county.

Mercer Co., A. W. Weir had nothing of interest to report.

Northumberland Co., A. O. Cawley said that rabies, hog-colera, and influenza was as numerous as common, but no move had been made to suppress them.

Schuylkill Co., J. W. Sallade made a detailed report of the local conditions in the county, bearing upon the condition of stock and numerous diseases, including parturient apoplexy in cows, which he attributed to the large use of brewers' grains.

Susquehanna Co., E. E. Tower said that they had had some hog-cholera, and a very peculiar disease among cattle during the last summer. It caused considerable loss in all parts of the county, and was investigated by the S. L. S. S. B., but the cause was not discovered. He had used injections of anthrax vaccine, which he believed had arrested the spread of the disease.

Venango Co., George B. Jobson had had considerable influenza, azoturia, and black-leg to treat. He also reported two outbreaks of rabies, in one of which a lady was bitten by a rabid dog and died six weeks after. Five children were also bitten by a dog with hydrophobia. They were sent to the New York City Board of Health and given the Pasteur treatment, which had proved satisfactory in each case. Dr. Pearson inquired if this outbreak was confined to Pennsylvania, and Jobson replied that the lady was supposed to have been bitten in Ohio.

Allegheny Co., Dr. Rectenwald reported for Allegheny Co., owing to the absence of J. Stewart Lacock, who is secretary for this county. He said they had had a peculiar form of influenza in horses. He had heard rumors of dogs with hydrophobia, but had seen no cases of this disease. Forage-poisoning is common, and tuberculosis is about as prevalent as usual. He had had several serious cases of azoturia. He had seen only two cases of osteo-porosis in the past year.

Dr. Jobson spoke again on the subject of hydrophobia, and referred to a goose which had been bitten by a rabid dog and later developed a well-marked case of hydrophobia.

The reports of delegates to veterinary medical associations were next made.

John W. Adams, George B. Jobson, and Otto Noack were the delegates to the American Veterinary Medical Association. As none of these gentlemen attended the meeting, there was no report. Dr. W. Horace Hoskins reported for this committee, and noted especially the improvement it had made in the past ten years.

(To be continued.)

